# Shoulder pain that is not shoulder pathology – a shoulder surgeon's guide to concomitant cervical spine and rotator cuff disease

**DOI:** 10.1016/j.xrrt.2026.100690

**Published:** 2026-02-06

**Authors:** Gregory R. Sprowls, Mitchell Parker, Thomas R. Denninger, Charles A.Thigpen, Adam Lutz, Stephan G. Pill, Timothy P. McHenry, Michael J. Kissenberth

**Affiliations:** a Steadman Hawkins Clinic of the Carolinas, Greenville, SC, USA; b Baylor College of Medicine, Temple, TX, USA; c Baylor Scott & White Health Dept. of Orthopedic Surgery, Temple, TX, USA; d ATI Physical Therapy, Greenville, SC, USA; e Institute For Musculoskeletal Advancement, Downers Grove, IL, USA; f Prisma Health Dept. of Orthopedic Surgery, Greenville, SC, USA; g Novant Health Orthopaedics & Sports Medicine, Greenville, SC, USA

**Keywords:** Shoulder pathology, Rotator cuff disease, Cervical spine, Surgical management, Neck pain, Rotator cuff repair

## Abstract

**Background:**

Rotator cuff and cervical spine disease are complex and nuanced conditions that commonly coexist, creating a challenging treatment paradigm.

**Methods:**

An algorithmic approach to diagnosis and management is developed in this study and illustrated through clinical vignettes.

**Results:**

The diagnostic and therapeutic challenges of concomitant shoulder and neck conditions may be simplified by this approach.

**Conclusion:**

Shoulder surgeons should seek cervical disease with a high index of suspicion and understand cervical conditions that may take surgical precedence to rotator cuff repair.

Rotator cuff and cervical spine conditions commonly coexist in patients who seek care for shoulder pain. The frequency that cervical pathology is overlooked in the treatment of shoulder conditions is difficult to quantify. The overlap of symptoms in patients with shoulder and cervical spine conditions, and the treatment of both regions to facilitate good patient outcomes, was described by Hawkins et al in 1990.^20^ More recently, others have refined the differential diagnoses and evaluation options for various shoulder and spine pathologies, emphasizing the difficulties in establishing a diagnosis and effective treatment plan.^19^^,^^42^ Poor sensitivities and specificities associated with elements of the correlative history, exam, imaging, and electrodiagnostic studies plague the diagnostic process.^6^^,^^13^^,^^15^^,^^51^ Rotator cuff and cervical spine disorders may even be causally linked so that successful treatment of one region requires effective treatment of the other.^25^^,^^29^ When concomitant cervical and rotator cuff disorders are correctly identified, establishing the predominate entity is often key in determining treatment priorities. This determination is frequently driven by provider preference and subspecialty, including the decision to proceed with shoulder or cervical spine surgery. Ultimately, successful treatment may require staged surgery in both regions. When surgical treatment is indicated in both regions, deciding which condition to address first is controversial. It has been suggested that, in the absence of cervical myelopathy, rotator cuff repair should be done first due to less perceived risk to the patient.^25^ In the instance of cervical myelopathy, it is recommended that cervical spine surgery proceed expeditiously to stabilize a patient's neurological function and prevent further deterioration.

The purpose of this review is to develop an evidence-based guide to aid the diagnosis and treatment of patients with concomitant rotator cuff and cervical spine pathology. An awareness of the broad differential diagnosis of disorders in these patients, including uncommon diagnoses, will allow for more uniform prioritization of evaluation and treatment options across orthopedic subspecialties.

## Diagnostic obstacles

### Overlapping symptoms

According to the most recent National Institutes of Health survey data, roughly 19% of adults 45 years and older have neck pain.^37^ Shoulder pain is the third most common musculoskeletal complaint, with a prevalence from 6.9% to 26%.^24^ Considerable muscular and neurologic anatomic overlap exists between the 2 regions, frequently resulting in vague subjective complaints and combined neck and shoulder symptoms. Similarly, rotator cuff muscle weakness and deconditioning from tear pathology could reasonably exacerbate shoulder girdle weakness caused by cervical origin, and vice versa. Patients who recognize the dual origin of their symptoms often endorse shoulder discomfort but also localize pain to the levator scapulae, neck extensors, and infraspinatus.^1^ The shoulder pain patient that has undergone prior neck surgery or spine consultation may have increased awareness of potential cervical contribution; however, most patients are unaware of the overlapping conditions. Usually patients present with one chief complaint, either shoulder or neck pain, and only after thorough history and examination are both areas found to be potentially pathological. One in 4 patients with cervical radiculopathy had painful shoulder impingement on physical examination.^5^ In addition, Zhang et al^55^ found rotator cuff pathology in 25% of patients with cervical disease over the age of 60. In their study, 11% of patients over 60 with newly diagnosed cervical disease went on to develop cuff pathology within 5 years. Distinguishing symptoms due to rotator cuff disorders, cervical spine disorders, and peripheral compression neuropathy can be difficult.^25^ Clinical indicators for rotator cuff disorders include positional shoulder pain at night. Arm pain that radiates distal to the elbow has been suggested to be specific for cervical radiculopathy; however, the reliability of this principle has been questioned in a recent study from Hagiwara et al.^19^ While considering the above factors, the clinician must also keep in mind less common causes of overlapping shoulder and neck pain (Table I).

**Table I:** Uncommon causes of overlapping neck and shoulder symptoms.

Uncommon causes of overlapping neck & shoulder symptoms	
Parsonage turner syndrome	Ankylosing spondylitis/DISH
Suprascapular neuropathy	Syringomyelia
Thoracic outlet syndrome	Amyotrophic lateral sclerosis
Scapular dyskinesis	Multiple sclerosis
Atraumatic sternoclavicular disorders	Complex regional pain syndrome
Cubital tunnel syndrome	Fibromyalgia
Carpal tunnel syndrome	Quadrilateral space syndrome

*DISH*, diffuse idiopathic skeletal hyperostosis.

### Imperfect exam maneuvers

To solve the problem of tandem shoulder and neck pain, thorough physical examination is a reasonable endeavor. Yet, existing clinical examination maneuvers for both spine and shoulder issues have proven to be imperfect tools.^17^^,^^43^^,^^47^^,^^48^^,^^51^^,^^52^ A battery of common cervical spine and shoulder tests have been found to have poor independent sensitivity and only moderate reliability in multiple studies.^17^^,^^43^^,^^47^^,^^48^^,^^51^^,^^52^ Using multiple examination maneuvers together as a “cluster” has been recommended for both fields, but few orthopedic specialists have the clinical acumen necessary to do this effectively for both the neck and shoulder. Spine and shoulder specialists often view common disease states differently, further leading to diverging clinical opinions or missed diagnoses.

### Nonspecific magnetic resonance imaging findings

Magnetic resonance imaging (MRI) of the cervical spine and/or shoulder is often used to confirm and direct the diagnosis process. However, the use of MRI cervical spine findings without correlation symptoms and physical examination is not recommended because of the relatively high prevalence of abnormal MRI findings in asymptomatic patients. These findings increase with age. No specific MRI findings were correlated with the clinical complaints of neck pain, hand function, or clumsiness in one analysis.^11^ In a study comparing cervical MRI findings in patients with and without neck and shoulder symptoms, there was considerable overlap in pathology found and only disc herniation correlated with neck pain.^45^ Boden et al^4^ reported that one-quarter of asymptomatic volunteers older than 40 years had either a disc herniation or foraminal stenosis. Nakashima et al^36^ also reported, in a study of asymptomatic volunteers, a 5.3% rate of spinal cord compression and a 2.3% rate of spinal cord myelomalacia, both increasing with age. Therefore, the presence of abnormal imaging alone is insufficient in determining that the cervical spine is the symptom generator. The key in the evaluation for possible cervical radiculopathy is the correlation of physical findings to MRI or computed tomography (CT) myelogram findings. This correlation is made even more difficult due to the variability of anatomical patterns of radiculopathy with only 54% of patients having a standard pattern of radiculopathy in a recent study by McAnany et al^33^ In this study, patients with a nonstandard radiculopathy pattern differed by 1.68 levels from the standard pattern.

Advanced imaging of the shoulder is beset by a similar lack of correlation with symptomatology. An equal prevalence of partial thickness rotator cuff tears, subacromial fluid, acromioclavicular (AC) joint changes, and biceps and labral pathology was found in one analysis of asymptomatic and symptomatic shoulders.^2^ Only full thickness tears and glenohumeral osteoarthritis were more commonly found in symptomatic shoulders.^2^ It is well known that patients with unilateral symptomatic rotator cuff tears have a high incidence of similar-appearing but asymptomatic cuff tears in the contralateral shoulder as well.^35^^,^^53^^,^^54^ Multiple studies have found high rates of intra-articular and labral pathology in asymptomatic hockey players, rock climbers, and overhead athletes.^10^^,^^18^^,^^46^

### Unreliable electrodiagnostic studies

Electrodiagnostic studies are useful to assess for peripheral compression neuropathy, with a high positive and negative predictive values for those disorders.^44^ However, these studies lack similar diagnostic value for cervical radiculopathy. According to a review from Marquardt et al,^31^ nerve conduction studies should not be routinely used to diagnose radiculopathy. These studies do not test C-type sensory fibers, which are commonly the only nerve fibers affected by compression radiculopathies and the reason why pain and paresthesias, rather than motor or true sensory symptoms, are typically encountered.^31^ In patients with severe compression, motor nerve conduction studies can sometimes be abnormal. However, this requires that more than 50% of motor axons be affected, which is rarely the case. Electromyography, or needle electrode examination, has a specificity ranging between 87% and 100% and may be helpful in the diagnostic workup.^31^^,^^49^ This electrodiagnostic study has poor sensitivity as significant motor axon damage to 2 different muscles of the same myotome but different peripheral innervation is required to obtain an abnormal response.^31^

Despite the limitations of electrodiagnostic studies, a survey of 402 spine surgeons found a poor general understanding of these tools with 20% of respondents reporting their routine use in 10%-30% of their patients.^23^ The participants reported that, when the studies are utilized, 45% of the time it is to distinguish which level is causing symptoms, a capability these studies lack.^23^

## Clarifying principles for diagnostic evaluation

Several well-constructed and thorough reviews have been published that outline an exhaustive list of both cervical spine and shoulder clinical examination maneuvers. Here, the authors have attempted to provide only those maneuvers felt to specifically aid in differentiating between cervical spine and shoulder disorders.^21^ There is large overlap in symptoms due to shoulder disorders and cervical spine disorders, and these symptoms can be similar. Patients often have trouble distinguishing between radiating pain and radiating neurologic symptoms but are generally effective at describing how far down the arm these symptoms travel. Shoulder-derived symptoms do not usually travel past the elbow,^42^ although shoulder pain associated with carpal tunnel syndrome and cubital tunnel syndrome has been reported.^19^ Shoulder region pain that is exacerbated by overhead activities can suggest a shoulder disorder is the origin of symptoms. It is common for patients with dysfunction due to cervical spine disease to have a mixed picture, with component of both radiculopathy and myelopathy. The signs and symptoms of cervical myelopathy are variable and often not recognized, which can be particularly vexing due to concerns for developing unrecoverable loss of function. Early presenting symptoms may include extremity paresthesias, loss of hand dexterity, and gait dysfunction, such as ataxia or spasticity. Extremity weakness and bowel and bladder dysfunction may be seen later in the disease progression. Whereas the historical view was that a majority of patients experienced stepwise functional decline, more recent studies describe a more variable course of gradual decline in most patients.^28^ Correlation between symptoms and physical findings is essential for diagnosis of shoulder and cervical spine disorders, and there are many possible components of an individual practitioner's physical examination.

### Rotator cuff strength assessment and rotational lag signs

Standard strength testing of isolated rotator cuff muscles should be performed as would normally be done. However, particular attention should be paid to isolate weakness to specific cuff muscles and correlative these assessments with the degree of actual tendon disease found on MRI. Profound subscapularis weakness with only a small upper border subscapularis tear, for instance, may indicate that nerve root dysfunction is the more likely cause. In the same vein, rotational lag signs (external rotation lag sign, Hornblower sign) without significant cuff atrophy on sagittal MRI suggests a neurological issue is present.

### Cervical spine

The general physical examination of the cervical spine begins with a clinical assessment of alignment and range of motion (ROM), followed by a graded motor and sensory examination of the upper and lower extremities, determining the distribution of any symptoms of numbness, weakness, or paresthesia, and noting any deficits that may correlate with cervical spine pathology. Some individuals may have significant variability in symptoms relative to “standard” anatomical distributions because of variability in brachial plexus anatomy, atypical spinal cord neuroanatomy, and peripheral nerve anastomoses.^33^

There are many provocative tests for cervical radiculopathy, including the Spurling test, cervical distraction test, shoulder abduction sign, and arm squeeze test.^47^ There are several different descriptions of the Spurling test, applying various combinations of neck extension, ipsilateral tilt, and rotation towards the affected arm, with or without an axial load applied manually to the head. The Spurling test is commonly performed by applying manual compression to the head with the patient's neck extended and rotated toward the symptomatic arm. A positive Spurling test aggravates the patient's radicular arm symptoms. Conversely, the cervical distraction test is positive if the patient's radicular arm symptoms are decreased or resolved by applying axial distraction to the neck. The shoulder abduction test, sometimes called the shoulder abduction relief test, is positive when radicular symptoms are alleviated by the patient lifting the symptomatic arm overhead or placing their hand on their head. Recently, the arm squeeze test was described to differentiate between arm pain due to cervical spine and shoulder pathology. It is performed by applying circumferential pressure, “squeezing,” the upper arm. A positive test, correlating with cervical radiculopathy, is an increase in pain on the visual analog scale (VAS) by 3 or more points. The variability of sensitivity and specificity of these physical examination tests were noted in a recent review by Thoomes et al^47^ (Table II).

**Table II:** Provocative test for cervical^47^ radiculopathy.

Test	Sensitivity	Specificity
Spurling test	0.98	0.89
Shoulder abduction test	0.47	0.85
Cervical distraction test	0.33	0.97
Arm squeeze test	0.97	0.97

Physical examination findings in the presence of cervical myelopathy are variable, and there is no single examination finding that is pathognomonic for the presence of myelopathy (Table III).^8^^,^^9^ As opposed to the multiple provocative tests for cervical radiculopathy, Lhermitte sign is the only commonly used provocative test for cervical myelopathy. It is used to describe the phenomenon of a transient electric shock sensation felt through the spine into the extremities with flexion and/or movement of the neck. Uchihara et al^50^ reported that Lhermitte sign has a high specificity for compressive myelopathy, 97%, but a very low sensitivity, 3%, which limits its clinical utility.

**Table III:** Physical examination for cervical myelopathy (adapted from Cook et al, 2009^8^).

Functional tests	
Hand grip and release	Rapid opening and closing of hands with normal at least 20 repetitions in 10 s
Finger escape	The inability to maintain small finger adduction with the fingers extended
Gait	Observation of gait pattern for ataxia or spasticity
Tandem gait	Inability to perform a toe to heel walk
Pathological reflexes	
Hoffman sign	Stabilizing the long finger proximal interphalangel joint and "flicking" the distal interphalangeal joint into a flexed position to elicit reflexive index finger and thumb flexion
Scapulohumeral reflex	Tapping the scapular spine with a reflex hammer to elicit reflexive shoulder abduction
Inverted supinator sign	Eliciting reflexes finger flexion with testing the brachioradials deep tendon reflex
Cross-hip adductor reflex	Eliciting contralateral hip addution by tapping the insertion of the hip adductors in the medial knee region
Babinski reflex	Eliciting great toe extension and lesser toe abduction by applying stimulation to the plantar aspect of the foot with the blunt end of a reflex hammer from the lateral heel to medial metatarsal region
Deep tendon reflexes	
Biceps	Tapping over the associated tendon to elicit a motor response, grading from 0 to 4: 0 = areflexic, 1 = hyporeflexic, 2 = normoreflexic, 3 = hyper-reflexic, 4 = elicits sustained clonus
Brachioradialis	
Triceps	
Patellar	
Achilles'	

Interestingly, the sensitivity and specificity of commonly performed physical examination tests are inversely proportional, an important consideration when evaluating a patient for myelopathy (Table IV).^22^ There are notable limitations in all of the specific physical examination tests for myelopathy. Rhee et al^40^ reported an absence of hyperreflexia and pathological reflexes in up to 21% of myelopathic patients. Hoffman sign has a positive predictive value that varies between 35% and 100%, depending on the study.^22^ Paul et al actually found in a group presenting with onset cervical stenosis symptoms, imaging findings positive for cord compression were proportionally greater for patients lacking a Hoffmann sign than those with a confirmed Hoffmann sign. Furthermore, multiple potential pathological findings, such as deep tendon hyperreflexia or the presence of pathological reflexes such as the scapulohumeral reflex, may be blunted or absent in some patients, such as patients with diabetes mellitus and peripheral neuropathy. Sometimes, the only correlative physical finding in myelopathic patients is the presence of an ataxic or spastic gait, with gait spasticity having a greater sensitivity compared to generalized gait dysfunction.^22^ There are 2 patterns of gait dysfunction in myelopathy: a “pure” ataxic gait with imbalance and inability to walk straight vs. a lack of coordination with gait, a “herky-jerky” gait pattern”.

**Table IV:** Sensitivity and specificity of pathological reflexes in cervical myelopathy (adapted from Jiang et al^22^).

Therefore, an aggregate of functional assessment, deep tendon reflex evaluation, and the presence of pathological reflexes has greater sensitivity and is more useful to determine if concerns for myelopathy exist. If so, diagnostic imaging such as MRI or CT myelography should be obtained to avoid unrecoverable functional loss from unrecognized and untreated cervical myelopathy.

### Diagnostic injections

There is good evidence that transient improvement of greater than 50% of radicular pain after a cervical transforaminal epidural steroid injection predicts successful cervical decompression surgery.^12^ On the other hand, Throckmorton et al^26^ expressed concerns for possible false-positive cervical selective nerve root blocks, especially at the C5 level, if there was “downstream” relief of pain due to a shoulder disorders mediated by the C5 sensory level.

## Recommendations for treatment

### Conservative management and physical therapy of the shoulder

**Figure 1:**
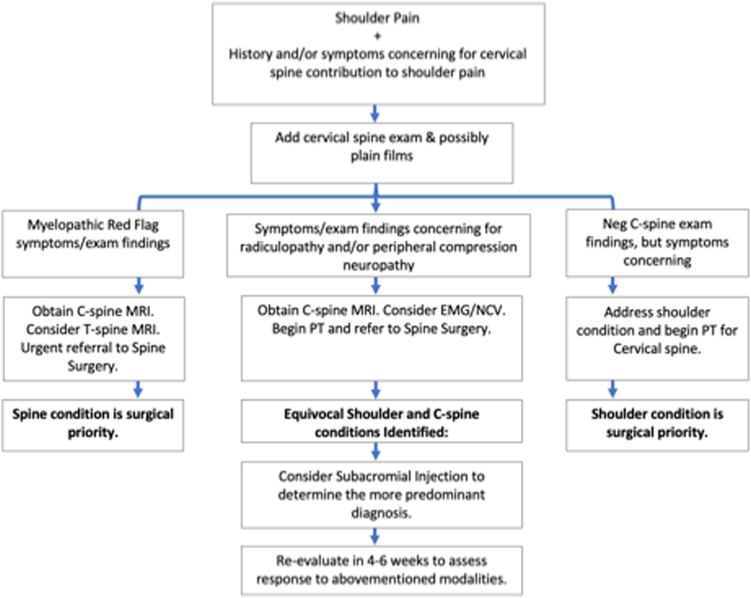
Algorithm for management of combined neck and shoulder pathology. *EMG*, electromyography; *MRI*, magnetic resonance imaging; *NCV*, nerve conduction velocity.

Rotator cuff tear progression often is difficult to predict. Factors associated with enlargement of the tear include advanced age, involvement of 2 or more tendons, and increasing symptoms. Even for full-thickness tears, nonsurgical treatment can be effective. A treatment plan should take into account the etiology, acuity, severity, and secondary maladaptations secondary to the pathology (Fig. 1). Pain control can include anti-inflammatory medications. Both naproxen and diclofenac have been shown to be superior to placebo in improvement of active abduction and function in those with full-thickness rotator cuff tears.^38^ Subacromial corticosteroid injections can be done acutely for diagnostic and therapeutic purposes, although it is not uncommonly used for refractory cases. Blair et al demonstrated improvement in pain and ROM in a randomized trial with triamcinolone and lidocaine vs. lidocaine alone, although there is contrasting literature demonstrating no improvement with corticosteroids. As always, the practitioner must weigh the potential side effects of using corticosteroids. There is stated efficacy in these injections allowing for participation in an exercise-based physical therapy program. Programs should be focused on strength and flexible deficits involving the shoulder.^28^ This can even include the trunk and lower extremity. Shoulder strengthening including scapular stabilization and rotator cuff strengthening exercises should be included. Stretching of the posterior capsule may improve the dysfunction of both glenohumeral and scapular motion. Kuhn et al found that rotator cuff tear progresses in about 50% of people at relatively slow rates. Progression of untreated rotator cuff tears showed no statistical difference in progression comparing symptomatic patients to those without symptoms.^27^ There is limited knowledge as to whether physical therapy increases rate of tear progression.

McClure et al^34^ discusses the Staged Approach for Rehabilitation Classification: Shoulder Disorders, which includes identifying the level of tissue irritability and identifying the level of impairments. For a patient that has a diagnosed rotator cuff injury, an irritability rating is applied and the appropriate intensity of physical stress and manual manipulation is applied, which is in line with known timing of tissue healing. There is a general consensus that therapeutic exercise as well as manual therapy techniques can decrease pain while improving active ROM. The Multicenter Orthopaedic Outcomes Network (MOON) Shoulder Group demonstrated nonoperative treatment using physical therapy is effective for treating atraumatic full-thickness rotator cuff tears in 75% of patients, although those with neck disorders were excluded from their study.^27^ There is a lack of literature for nonoperative treatment of cervical myelopathy. Surgical intervention should be strongly considered in these patients.

### Conservative management and physical therapy of the cervical spine

For patients presenting with either primary or concomitant cervical findings, physical therapy may also play an important role in their plan of care. Current Clinical Practice Guidelines recommend a combination of manual therapy and exercise to improve pain, function, and quality of life in patients with both nonspecific localized neck pain and radiating symptoms.^3^ Many of these exercises overlap with those used for shoulder rehabilitation, as improved performance of the shoulder girdle and postural muscles is also beneficial for patients with neck pain. In addition, motor control exercises targeting the deep cervical flexors and extensors help enhance dynamic support of the cervical spine. Manual therapy interventions, including mobilization and manipulation of the cervicothoracic spine and adjacent soft tissues, may contribute to reduced pain and increased ROM. Mechanical or manual cervical traction can also be considered in patients experiencing radiating upper-extremity symptoms of cervical origin.^41^ While conservative care has demonstrated value in managing mechanical neck pain, there remains a lack of literature supporting nonoperative treatment for cervical myelopathy, and surgical intervention should be strongly considered for these patients.

### Arthroscopic rotator cuff repair

Arthroscopic rotator cuff repair (ARCR) of full-thickness tears demonstrates improved outcomes. Arthroscopy has evolved to improve in the complications of the open repair: deltoid avulsion, infection risk, poor visualization, and increased pain. Biomechanically advantageous repair constructs, suture materials, anchor technology, and biological augmentation have been developed to improve tendon repair integrity; however, achieving tendon healing remains a challenge. Nonetheless, ARCR has been shown to provide excellent clinical outcomes and return of function in the majority of patients.^14^ Mather et al demonstrated operative treated patients with atraumatic rotator cuff tears return to work sooner and incur less cost burden when compared to patients treated nonoperatively. These outcomes do not diminish with midterm and long-term follow-up.^32^ Piper et al^39^ found statistically significant differences favoring surgery in atraumatic full-thickness rotator cuff tears in both Constant and VAS scores after 1 year. However, both values were below the minimal clinically important differences for the Constant and VAS scores. In regard to rotator cuff tear progression, there is abundant literature that demonstrates natural tear progression in 42%-82% of patients as time passes.^30^ There is limited high-quality data that demonstrates surgery may limit progression of rotator cuff, and further studies are needed to explore if smaller tears in younger patients may benefit from surgical intervention to prevent further progression.

## Cervical spine surgery

Although cervical degenerative disease can frequently be managed nonoperatively, surgical intervention may be a reasonable option for persistent or progressive radiculopathy and recommended for progressive myelopathy. There are many specific surgical options beyond the scope of this review. These range from single level to multilevel decompression and fusion surgeries that may be performed through anterior, posterior, or the combination of approaches. Motion-sparing techniques, including cervical laminoplasty or total disc arthroplasty, are also potential options for many patients, depending on the specific symptoms and pathological anatomy.^16^ In general, the goal of surgery for cervical radiculopathy is to alleviate radicular pain and paresthesia, whereas the goals of surgery for myelopathy are more limited. Patients are often advised that the goal of surgery for cervical myelopathy is to stabilize their neurological function, although some improvement is common. Return to normal function after surgery for cervical myelopathy is infrequent.^7^

### Clinical vignette #1 (shoulder symptoms attributable to cervical pathology)

**Fig. 2:**
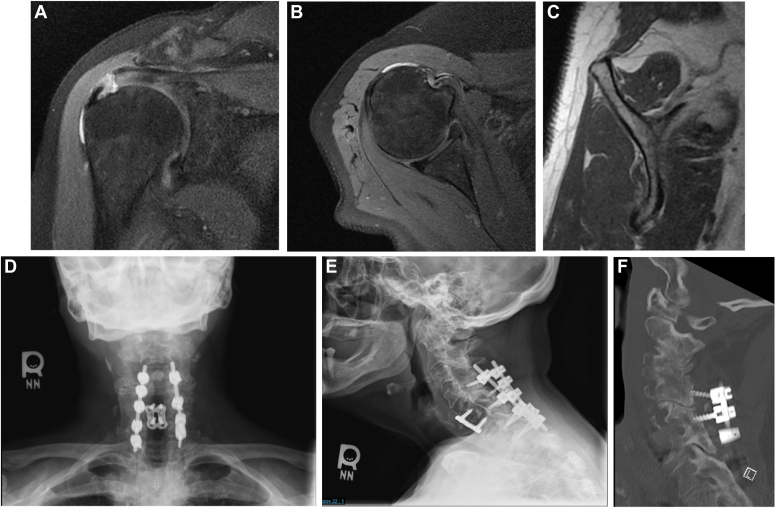
(**A-C**) MRI and radiographic images from clinical vignette #1. (**D-F**) Radiographic images from clinical vignette #1. *MRI*, magnetic resonance imaging.

A 73-year-old female presents to the shoulder and elbow clinic with worsened chronic right shoulder pain and severely increased weakness for the past 7 months, having received a subacromial corticosteroid injection with near-complete relief for only 3 days. The pain was severe, localized to the shoulder, but also extending down the arm, past the elbow, into the palmar long fingertip. She has a past medical history of ischemic cardiomyopathy and 50–pack-year smoking history. Plain films of the right shoulder were normal. MRI of the right shoulder was significant for supraspinatus tendinopathy with a 2-cm full-thickness tear without significant retraction, subacromial bursitis, AC joint arthrosis, degenerative labral changes, and mild glenohumeral arthritis (Fig. 2 *A*–*C*). There were Goutallier grade 2 fatty atrophy to the supraspinatus muscle, 0 to the infraspinatus, and 1 to the subscapularis muscle. On physical exam of her right shoulder, she had 10 degrees of active flexion and external rotation and internal rotation to the hip, passive ROM to 170 degrees of forward flexion. She had a positive empty can, external rotation lag sign, belly press, Neer, Hawkins, Obrien, and Speed test. She had 2/5 strength to all rotator cuff tendon testing, as well to biceps and deltoid muscle testing. She complained of numbness to the palmar long fingertip.

Upon further questioning, she revealed a past surgical history significant for C5-C6 anterior cervical discectomy fusion (ACDF) 9 years prior. Pre-operative imaging was significant for cervical myelomalacia with cord compression, and pre-operative symptoms included bilateral extremity numbness, tingling, and pain down to the hands (Fig. 2 *D*–*F*). She experienced relief for 7 years but then developed gait disturbance, increasing numbness, tingling, and pain to bilateral extremities, for which she underwent subsequent C4-T1 posterior decompression and fusion. This surgery resulted in symptom improvement, but residual neck pain and occasional radiation to the bilateral shoulders and upper posterior arms.

Symptoms extending past the elbow and a surgical history of multiple cervical surgeries led to a more in-depth history taking. Upon more questioning, she denied gait disturbance, bowel or bladder incontinence, or loss of dexterity of her hands. She states the shoulder pain is worse on her right, but she does have mild symptoms in her left shoulder as well. A cervical physical exam was completed, which was significant for limited ROM of the neck with 25 degrees of rotation, 40 degrees of neck flexion and extension. She had a negative Lhermitte sign and Hoffman sign and lacked any myelopathic signs. With a high index of suspicion for cervical etiology of symptoms, a repeat cervical x-ray was obtained the same day, which demonstrated screw pullout of the right C4 and C5 articular pillar screws. CT of the cervical spine demonstrated further hardware loosening. She was placed in a C-collar and referred to her spine surgeon and physical therapy to begin deltoid strengthening. The patient underwent removal of hardware and revision C4-T1 arthrodesis 3 months later, with improvement of right shoulder pain but continued sensory symptoms to the bilateral hands.

### Clinical vignette #2 (cervical myelopathy addressed surgically prior to arthroscopic rotator cuff repair)

**Fig. 3:**
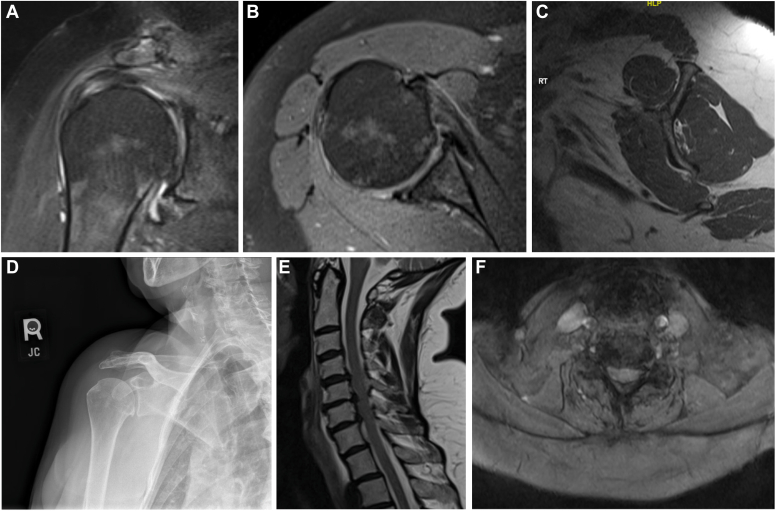
(**A-D**) Imaging from clinical vignette #2. (**E** and **F**) MRI from clinical vignette #2. *MRI*, magnetic resonance imaging.

A 69-year-old female with a past medical history of rheumatoid arthritis, for which she takes 4 different disease-modifying antirheumatic drugs, presented with right shoulder pain ongoing for 3 years that has become acutely over the last 3 months. She has undergone multiple injections from a previous practitioner with diminishing result. She has significant difficulty with overhead activities, diffuse, vague shoulder pain, and weakness with attempted lifting. On physical exam, she has diffuse tenderness about the shoulder and active forward flexion to 90°, external rotation 25°, and internal rotation to the hip. Passive forward flexion was 130° with severe pain, and passive external rotation in adduction was 35° with pain. Weakness was noted to supraspinatus, posterior cuff musculature, and normal subscapularis testing. Right shoulder plain films reveal mild loss of glenohumeral joint space, and MRI of the right shoulder revealed a supraspinatus partial tear, subscapularis tendinosis, and upper border tear with biceps subluxation at the top of the groove, subacromial bursitis, and moderate AC joint arthritic changes (Fig. 3 *A*–*D*).

Upon further questioning, she also complains of neck pain, numbness, and tingling to her bilateral upper extremities to the dorsal long and index fingers, trouble with grip strength, and gait issues. She has difficulty with balance and requires the use of a wheelchair due to frequent falls. Plain films of the cervical spine were obtained that day, demonstrating mild multilevel degenerative changes, most concentrated at the C5-6 level. Routine MRI was ordered to evaluate for disc disease.

She underwent a diagnostic and therapeutic steroid injection to the right glenohumeral joint and was sent to physical therapy for deltoid strengthening. A few weeks later she had worsened neck pain, upper extremity numbness, tingling, and balance issues. She presented acutely to the emergency department, where an urgently cervical MRI demonstrated central disc protrusions at C5-7 with severe spinal cord compression (Fig. 3 *E* and *F*). She underwent prompt ACDF of C5-C6 and C6-C7 and returned to the shoulder and elbow clinic 3 months post-operatively. She reported significant improvement of her neck pain, numbness and tingling, and balance issues. She was no longer confined to a wheelchair. Her main issue was persistent right shoulder pain of a different quality, more concentrated to the anterior proximal biceps region of the shoulder. She had continued formal deltoid strengthening with physical therapy throughout this period and had regained active forward flexion to 160°, external rotation to 60°, and internal rotation to L5. With the improvement in her shoulder ROM, she now noticed specific mechanical clicking and catching to the anterior shoulder. Therefore, four months after her ACDF, she underwent right shoulder arthroscopy with supraspinatus débridement, repair of upper border subscapularis, biceps tenodesis, and débridement with complete resolution of her shoulder pain and mechanical symptoms.

### Clinical vignette #3 (diagnostic corticosteroid injections used to direct surgical care)

**Fig. 4:**
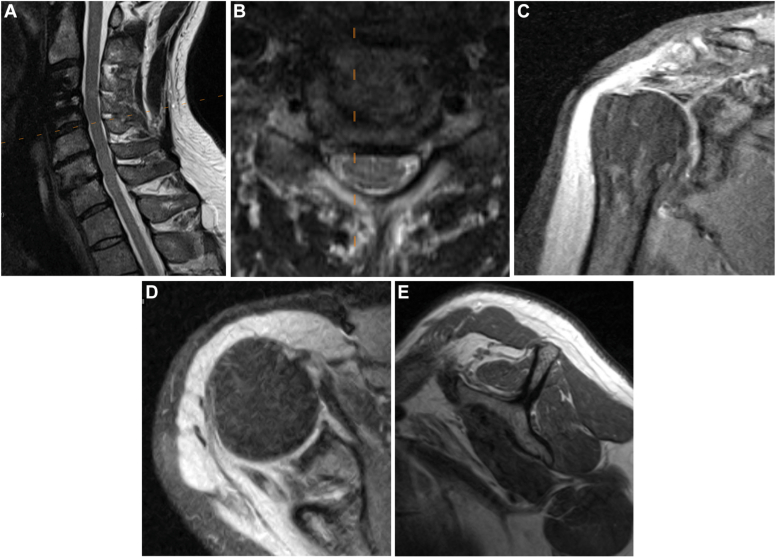
(**A-E**) MRI from clinical vignette #3. *MRI*, magnetic resonance imaging.

A 43-year-old male presented with right worse than left shoulder pain after a car accident sustained 4 months prior. He sustained an anterior longitudinal ligament disruption found on MRI cervical spine with cord effacement and abnormal signal at C3-C4 (Fig. 4 *A*–*E*). He subsequently underwent ACDF to levels C3-C4. His pre-operative symptoms included severe weakness to the bilateral upper extremities with inability to raise past 45° of forward flexion. After recovering from the cervical surgery, he regained near-full function of the left shoulder. He regained near-full active motion to the right shoulder (170° forward flexion, 45° external rotation, internal rotation to L5) but continued to experience persistent, severe right shoulder weakness. He demonstrated no strength against minimal resistance in testing of all cuff tendons, deltoid, and biceps muscle groups. He was noted to have positive empty can, belly press, Neer, Hawkins, Obrien, and Speed tests. External rotation lag sign was negative. Sensation was intact to light touch of the hand, but he had paresthesias and slightly decreased sensation over the axillary nerve distribution to the lateral deltoid. He denied gait disturbance, bowel or bladder dysfunction, or decrease in fine motor skills. Plain films of the shoulder were unremarkable. MRI of the shoulder revealed cuff tendinosis with a small, near-full-thickness tear of the anterior supraspinatus, an upper-border subscapularis tear, and medial biceps subluxation. Electromyography demonstrated changes consistent with chronic bilateral C5 radiculopathy. To further investigate, repeat MRI of the cervical spine was obtained, which revealed small disc protrusions and severe foraminal stenosis from levels C3-C7.

To elucidate how much of his shoulder symptoms may be attributable to the anatomic abnormalities found in the shoulder, a diagnostic subacromial corticosteroid injection was performed. He experienced two days of moderate pain relief but no improvement in his functional motion or strength. His pain returned on day 3 after the injection. He was sent back to his spine surgeon for consideration of revision surgery. Conservative management and physical therapy for cervical radiculopathy was utilized, and he had gradual improvement in symptoms and overall shoulder function.

### Clinical vignette #4 (unknown cuff healing consequences of untreated cervical spine disease)

**Fig. 5:**
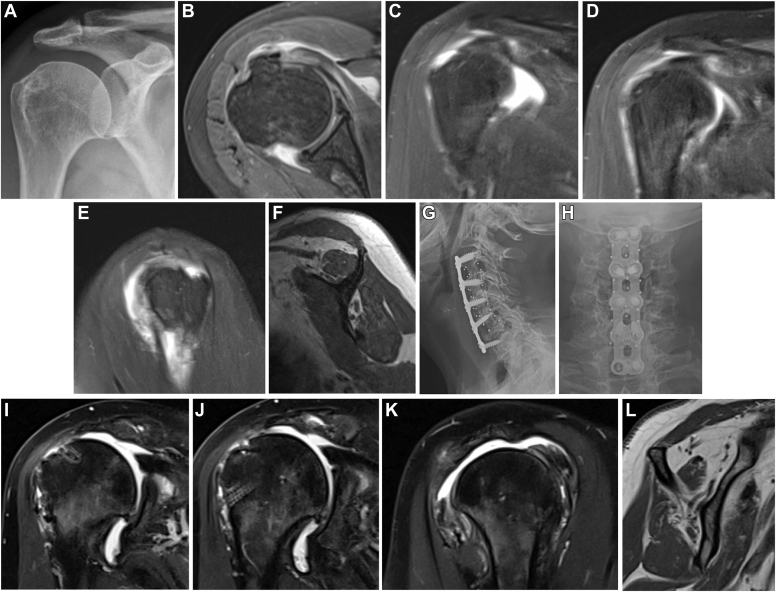
(**A-E**) Pre-operative images from clinical vignette #4. (**G-H**) Post-operative radiographic images from clinical vignette #4. (**I-L**) Post-operative MRI images from clinical vignette #4. *MRI*, magnetic resonance imaging.

A 66-year-old female presented with severe right shoulder that began after she fell and heard a pop 3 months prior. She had no shoulder symptoms prior to the injury and now has pain and weakness with overhead activities, pain at night, and anterior based shoulder discomfort. She was not amenable to subacromial steroid injection and exhausted nonsurgical modalities. Pre-operative examination included imaging (Fig. 5 *A*–*E*). It revealed active forward flexion of 90°, external rotation of 35°, and internal rotation to L1. She exhibited significant weakness to supraspinatus and subscapularis testing and mild weakness to posterior cuff testing.

Standard, transosseous-equivalent, double-row ARCR was performed of a medium-sized (2 cm × 2 cm) supraspinatus tear with retraction to the mid humeral head. Single-row subscapularis repair, arthroscopic suprapectoral biceps tenodesis, and acromioplasty were performed as well. The patient did well post-operatively until the 9-month mark, when she had insidious recurrence of pain, weakness, and ROM deficits. She also complained of worsening neck pain, stiffness, and radicular symptoms down the right arm, past the elbow. Upon identification of these symptoms, a thorough cervical spine exam was performed with negative Lhermitte, Spurling, and shoulder abduction signs. There were no signs of myelopathy or severe sensory or gross motor deficits to the upper arm and hand. There was weakness to supraspinatus, posterior cuff, and subscapularis testing, and positive external rotation lag sign.

Further questioning revealed a history of multiple cervical spine surgeries years prior and persistent chronic neck pain since. Plain films (Fig. 5 *G* and *H*) were obtained and she was referred to her spine surgeon. Repeat shoulder MRI (Fig. 5 *I*–*L*) was ordered, demonstrating failure of the repair, massive 3 tendon tear with retraction to the glenoid, and significant progression of fatty atrophy. After failing an additional attempt at conservative management and therapy for the shoulder, she underwent successful reverse shoulder arthroplasty.

## Conclusion

Rotator cuff and cervical spine pathology commonly coexist. When the clinical history and physical exam point to combined pathology, it can be a challenge to determine which pathology should be addressed first. If cervical pathology is suspected to contribute to shoulder pain via a thorough physical exam and history, we recommend obtaining cervical spine radiographs and obtaining cervical spine MRI or CT myelogram imaging if there are persistent symptoms of radiculopathy or myelopathy. MRI or CT myelogram imaging of the thoracic spine should also be considered, although thoracic myelopathy is less frequent. Expeditious referral to an orthopedic spine surgeon or neurosurgeon is recommended for signs, symptoms, and imaging results concerning for progressive myelopathy. Persistent radicular symptoms should prompt referral to a spine surgeon after physical therapy treatment in the setting of progressive and/or functionally limiting motor weakness. If equivocal shoulder and C-spine conditions are suspected and neither condition has a more urgent surgical indication, subacromial injection and/or targeted cervical spine injections should be considered. If both conditions are projected to require elective, nonurgent surgical intervention, the rotator cuff condition should generally be addressed first. However, if there is evidence of myelopathy, severe radiculopathy, or profound deltoid or biceps weakness, cervical spine surgery should proceed first.

The most pivotal takeaway for shoulder-treating surgeons is to maintain a low threshold to inquire about neurologic symptoms to the upper extremity and to screen for prior cervical spine procedures and current symptoms of cervical disease. As demonstrated in the included vignettes, failing to do so might lead to omission of proper cervical spine examination and possibly an ill-guided shoulder operation. We recommend against routine, rote cervical spine examination for every shoulder pain patient to avoid adding potentially unnecessary measures to already overburdened clinic encounters. However, shoulder surgeons should seek cervical disease with a high index of suspicion and utilize the presented clinical cues to direct the differential diagnosis.

Diagnosing a coexisting cervical spine condition is clearly important in severe disease states to render timely care and prevent devastating neurologic consequences. Potentially more vital than previously recognized are the effects of less salient cervical disease states on healing rates and patient outcome measures after routine ARCR. As it stands currently, the recommendation to proceed with rotator cuff surgery first is not evidence-based or driven by outcome data. To further refine the management algorithm of combined shoulder and cervical spine conditions, future studies should aim to better elucidate the effects of unaddressed cervical cord or nerve root impingement on the success of rotator cuff repair.

## Disclaimers:

Funding: This project did not receive any funding.

Conflicts of interest: The authors, their immediate family and the research foundation associated with which they are affiliated did not receive any financial payments or other benefits from any commercial entity related with the subject of this article.
